# A functional polymorphism in the prodynorphin gene affects cognitive flexibility and brain activation during reversal learning

**DOI:** 10.3389/fnbeh.2015.00172

**Published:** 2015-07-03

**Authors:** Mikhail Votinov, Juergen Pripfl, Christian Windischberger, Ewald Moser, Uta Sailer, Claus Lamm

**Affiliations:** ^1^Social, Cognitive and Affective Neuroscience Unit, Department of Basic Psychological Research and Research Methods, Faculty of Psychology, University of ViennaVienna, Austria; ^2^Department of Psychiatry, Psychotherapy and Psychosomatics, RWTH Aachen UniversityAachen, Germany; ^3^MR Center of Excellence, Center for Medical Physics and Biomedical Engineering, Medical University of ViennaVienna, Austria; ^4^Department of Psychology, University of GothenburgGothenburg, Sweden

**Keywords:** prodynorphin, opioid system, reversal learning, functional MRI, genetics

## Abstract

Whether the opioid system plays a role in the ability to flexibly adapt behavior is still unclear. We used fMRI to investigate the effect of a nucleotide tandem repeat (68-bp VNTR) functional polymorphism of the prodynorphin (PDYN) gene on cerebral activation during a reversal learning task in which participants had to flexibly adapt stimulus-response associations. Past studies suggested that alleles with 3 or 4 repeats (HH genotype) of this polymorphism are associated with higher levels of dynorphin peptides than alleles with 1 or 2 repeats (LL genotype). On the behavioral level, the HH group made more perseverative errors than the LL group. On the neural level, the HH group demonstrated less engagement of left orbitofrontal cortex (lOFC) and cortico-striatal circuitry, and lower effective connectivity of lOFC with anterior midcingulate cortex and anterior insula/ventrolateral prefrontal cortex during reversal learning and processing negative feedback. This points to a lower ability of the HH genotype to monitor or adapt to changes in reward contingencies. These findings provide first evidence that dynorphins may contribute to individual differences in reversal learning, and that considering the opioid system may shed new light on the neurochemical correlates of decision-making and behavioral regulation.

## Introduction

The ability to inhibit or flexibly modify established stimulus-response-associations is vital for all species in order to survive in a changing environment. For instance, previously rewarded behaviors may become punished, requiring behavioral adaptations. Empirically, the ability of re-learning behaviors as a response to changes in their reward properties can be evaluated by the well-established probabilistic reversal learning task (e.g., Cools et al., 2002). Previous lesion, neuroimaging and pharmacological human and animal studies underlined the role of brain structures such as the striatum, thalamus, anterior cingulate cortex (ACC), lateral orbitofrontal cortex (OFC), and anterior insula (aInsula)/ventrolateral prefrontal cortex (VLPFC) in reversal learning mechanisms (Duncan, 2001; Cools et al., 2002; Fellows and Farah, 2003; Kringelbach and Rolls, 2003; Clark et al., 2004; Hornak et al., 2004). On a neurochemical level, many studies stressed the regulatory role of the dopamine system in adaptive behavior (Clark et al., 2004; Robbins and Arnsten, 2009; Groman et al., 2013). Studies using pharmacological interventions and genetic polymorphisms of dopamine receptors indeed suggest that both dopamine deficiency and overstimulation by dopamine is related to impairments in reversal learning (Smith et al., 1999; Cools et al., 2006; Pessiglione et al., 2006; Cohen et al., 2007; Jocham et al., 2009). In addition, serotonin seems to play a role in reversal learning, as its depletion was associated with impaired reversal learning (Rogers et al., 1999; Clarke et al., 2007), for review see (Homberg, 2012).

There is, however, a distinct lack of knowledge about the role of the opioid system in reversal learning mechanisms. Prodynorphin (PDYN), the gene coding for the dynorphin opioid peptides, is a strong candidate for influencing a range of neuronal circuits, including reward pathways. In the human brain the PDYN gene is predominately expressed in the cingulate cortex, the amygdala, the dentate gyrus, and the striatum (Hurd, 1996). Dynorphins show high affinity particularly to the κ-opioid receptor and can regulate dopamine release in the striatum and prefrontal cortex (Di Chiara and Imperato, 1988b; Steiner and Gerfen, 1998; Margolis et al., 2006). It may also inhibit the release of glutamate, GABA (Hjelmstad and Fields, 2001, 2003), and serotonin (5-HT) (Pinnock, 1992; Tao and Auerbach, 2005). In terms of behavior, various studies across different species have demonstrated effects of dynorphins on memory, learning, and cognitive functions (Colombo et al., 1992; Yakovleva et al., 2007; Kölsch et al., 2009; Tejeda et al., 2012; Kuzmin et al., 2013; Bilkei-Gorzo et al., 2014). Dynorphins may also contribute to aberrant habit formation in humans, as shown by their link to drug consumption and addiction (Everitt et al., 2001; Hyman and Malenka, 2001; Shippenberg et al., 2007). For these reasons, dynorphins may be expected to affect reversal learning.

However, there is as of yet no evidence about how polymorphisms in the PDYN gene modify learning and adaptive behavior in healthy human participants. One such functional polymorphism consisting of 1–4 repeats of a 68-bp element in the promoter region of the PDYN gene was first described by Zimprich et al. (2000). Several *in-vivo* and *in-vitro* studies demonstrated that alleles with 3 or 4 repeats of this variable nucleotide tandem repeat (VNTR) are associated with higher levels of dynorphins expression than alleles with 1 or 2 repeats (Zimprich et al., 2000; Nikoshkov et al., 2008; Babbitt et al., 2010). More recently, though, another study suggested that the alleles with 1 or 2 repeats may be associated with higher dynorphins availability and/or potency (Rouault et al., 2011). This controversy notwithstanding, these studies provide evidence that variation in 68-bp repeat number is functionally significant for the availability of dynorphins (Zimprich et al., 2000; Nikoshkov et al., 2008; Babbitt et al., 2010).

We assessed the neuronal processes during reversal learning in participants showing differences in the PDYN 68 bp VNTR polymorphism, by using BOLD-based functional magnetic resonance imaging (fMRI). Our general objective was to explore the role of the DYN/KOPr opioid system for the ability to reverse previously learned rewarding activities, as this might be the key to understanding why dysregulation of this system is associated with drug abuse. Along these lines, in a recent review by Izquierdo and Jentsch (2012), reversal learning was reported to be impaired in individuals affected by addictions and it was proposed that reversal learning tasks may serve as a diagnostic tool for investigating the neural mechanisms of reward seeking and reward consumption behavior (Izquierdo and Jentsch, 2012).

## Methods

We screened 286 healthy Caucasian volunteers with no history of psychiatric or neurological disorders or contraindications for high-field MRI scanning for their genotype in the PDYN 68-bp VNTR polymorphism. All participants signed informed consent and the study protocol was approved by the ethics committee of the Medical University of Vienna.

### Genetic analyses

DNA was determined based on saliva samples collected using a self-collection kit designed for the collection and storage of DNA (Oragene DNA, DNA Genotek, Ottawa, Canada). A commercial kit (Qiagen, Hilden, Germany) was used for DNA extraction. PDYN genotyping was performed according to established procedures at the DNA laboratory of the Department of Neurology of the Medical University of Vienna. In short, purified DNA was diluted into a PCR reaction mix consisting of 20 mM Tris-HCl (pH 8.8), 50 mM KCl, 1.5 mM MgCl2, deoxynucleotide triphosphates each at 0.4 mM, 10 pmol of each primer, and 0.6 U of Taq polymerase in a total volume of 30 μl. Amplification conditions were 30 s at 94°C, 45 s at 62°C, and 45 s at 72°C for 30 cycles using the following primers, which flank the entire promoter region: upstream (P1), 5′-AGC AAT CAG AGG TTG AAG TTG GCA GC; and downstream (P2), 5′-GCA CCA GGC GGT TAG GTA GAG TTG TC. The amplification products were resolved on a 2.5% agarose gel stained with ethidium bromide.

The allelic distribution of the genotype of interest PDYN (LL, LH, HH) was in Hardy–Weinberg equilibrium, χ^2^_(2)_ = 1.39, *p* = 0.5. This indicates that genotype frequency fits a predictable binomial distribution calculated from allele frequencies and conditions of population equilibrium are met (random mating and negligible mutation).

### Participants

Based on Zimprich et al. (2000), groups were designated based on the number of tandem repeats in the PDYN gene promoter. Alleles with 1–2 repeats were termed L alleles, and alleles with 3–4 repeats were termed H alleles, because H alleles in their study produce greater expression of PDYN than L alleles (see however, Rouault et al., 2011). This resulted in three genotypes, LL, LH/HL, and HH. The distribution of genotypes in the screening sample was: HH—142 participants, LH/HL—113, LL—31. We invited 25 participants from the HH and LL groups to the fMRI experiment. The groups were matched for age and gender prior to fMRI experiments. Due to lack of compliance (2), technical problems with stimulus presentation software (3), or missing data (2), a total of seven participants were excluded from the analyses. The final sample included 22 participants in the LL group and 21 in the HH group (see Table 1). The mean age for LL was 23.6 ± 1.4 (mean ± SE) years and for HH 23.04 ± 0.8 [difference not significant, *t*_(df)_ = 41, *p* = 0.72]. In addition, the groups were matched for alcohol, coffee, tobacco, and energy drinks use. For this purpose, they were asked to indicate on a 4-point scale how many times per week they drink (alcohol, coffee, and energy drinks) or smoke (0, not at all; 1, 1 time per week; 2, 1–3 times per week; 3, every day) (see Results and Table 2). All participants were drug free, as determined by a drug test for opiate, amphetamine, and cannabinoid substance use applied prior to scanning (Dip-Test MULTI 5/1, Dipro med, Austria).

**Table 1:** **Demographic table**.

**Genotype groups**	**Total number**	**Gender (Male/Female)**	**Age (mean ± SEM)**
LL	22	10/12	23.6 ± 1.4
HH	21	10/11	23.04 ± 0.8

**Table 2:** **Consumption of alcohol, tobacco, coffee, and energy drinks**.

**Genotype groups**	**Total number**	**Smoking (mean ± SEM)**	**Alcohol (mean ± SEM)**	**Coffee (mean ± SEM)**	**Energy drinks (mean ± SEM)**
LL	22	0.68 ± 0.21	1.22 ± 0.16	1.95 ± 0.23	0.59 ± 0.12
HH	21	0.61 ± 0.21	1.19 ± 0.16	1.66 ± 0.23	0.52 ± 0.13

*All p > 0.38*.

Participants filled in the BIS/BAS scale (Carver and White, 1994) which assesses sensitivity of the behavioral inhibition and approach systems (i.e., sensitivity to reward and punishment) and the Barratt Impulsiveness Scale (BIS11) which assesses impulsiveness (Patton et al., 1995). Each participant received a 30 euro participation fee.

### Task design

**Figure 1:**
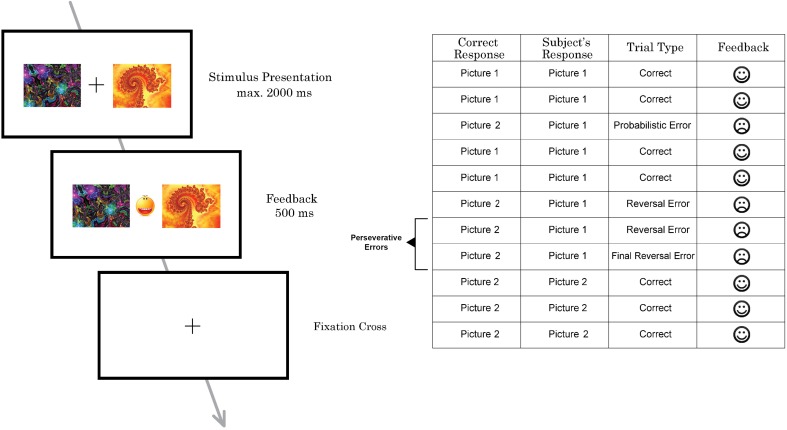
**Schematic illustration of one trial of the probabilistic reversal task performed by subjects in the MRI scanner (left) and part of a trial sequence with different types of errors (right)**.

The probabilistic reversal learning task which was developed and used in previous neuroimaging studies by Cools et al. (2002) was replicated and applied in this study (Figure 1), but with a lower number of trials. On each trial, participants had to select one of two abstract fractal pattern stimuli presented, of which one was associated with a correct response and the other one with an incorrect response. Participants had to find and choose the correct stimulus by selecting the pattern via pressing a button on a response box. Immediately upon button press, either a happy face, indicating a correct response, or a sad face, indicating an incorrect response, were presented. Participants thus could learn the correct stimulus-reward contingency—i.e., which stimulus was rewarded. The reward rule, however, changed intermittently so that the other stimulus (fractal pattern) became correct, requiring participants to adapt their response accordingly. To increase task difficulty and to prevent the task from being deterministic, probabilistic errors were interspersed, occasionally indicating that a wrong response was entered although the correct stimulus had been chosen. Participants were instructed to only start switching to the other pattern when they were sure that the reward rule had changed.

Participants performed two sessions with 100 trials each. The task was not adaptive and reversal of the stimulus–reward contingency occurred after 10–15 trials (including probabilistic errors) in a random pattern (see Cools et al., 2002 for details). To prevent subjects from adopting a strategy such as always reversing after two consecutive errors, probabilistic negative feedback was given on two consecutive trials twice during each task session. The number of probabilistic errors between each reversal varied from 0 to 2. In total, 12 probabilistic errors were randomly implemented per session.

Stimuli were presented for 2000 ms. Feedback was presented immediately after the response in-between the two pictures for 500 ms. If the participant did not make a choice before 2000 ms had elapsed, a “too late”-message was presented on the screen. All participants, before entering the scanner, had taken part in 20 practice trials to familiarize them with the task and to minimize practice effects during the experiment.

### Analysis of behavioral data

We counted the overall number of perseverative errors and number of spontaneous errors for the two fMRI sessions. Perseverative errors were defined as errors (except the 1st one) which subjects committed after the reversal. Spontaneous errors were defined as errors when subjects shifted their response during a set of continuous correct trials, which may indicate failure of attention.

Additionally, we calculated the response time in the trials following perseverative errors (except for the first wrong trials in a sequence), following correct responses (except for the first correct trials in a sequence), and response time for the first trials after a final reversal errors (later termed “final errors”), which are errors followed by a switch to the correct response in the immediately following trial. First trials were excluded from the sequences of correct and wrong answers, because when subjects respond to the first trial in the sequence, they do not know whether the response is going to be correct or wrong. The 1st correct trials in a correct sequence after final reversal errors were analyzed separately, to estimate how much time subjects spend to decide to switch the strategy.

Group differences for number of errors were analyzed using repeated-measures ANOVA with type of errors as within-subjects factors (2 levels) and genotype groups (LL, HH) as between-subjects factor. The same analysis was applied to the data about level of consumption of alcohol, coffee, tobacco, and energy drinks and reaction time (RT). Here, the within-subjects factors were type of substance (4 levels) and type of RT (3 levels) and genotype groups (LL, HH) was the between-subjects factor.

During the analyses of the BIS/BAS and BIS11 scales the respective subscales (4 and 3 levels consequently) were used as within-subjects factors and genotype groups (LL, HH) as between-subjects factor. If the sphericity assumption was violated (significant results in Mauchly's test of sphericity), degrees of freedom were corrected using Greenhouse-Geisser estimates of sphericity. Significance was evaluated at *P* < 0.05. *Post-hoc* tests with Bonferroni correction for multiple comparisons were applied. All data are reported as means ± SE.

### MRI scanning

MRI scanning was conducted on a 3 Tesla TIM Trio whole body scanner (Siemens Medical Solutions, Germany), using the manufacturer's 32-channel head coil, at the MR Center of Excellence, Medical University of Vienna. Functional images were obtained with a single-shot echo planar imaging (EPI) sequence, with the following image acquisition parameters: repetition time (TR) = 1800 ms, echo time (TE) = 38 ms, flip angle (FA) = 72°, 294 whole-brain volumes (matrix size 128 × 128, FoV = 190 × 190 mm^2^, 3 mm slice thickness). For anatomical registration, we obtained high-resolution 3D T1 anatomical images after the fMRI session (MPRAGE, magnetization prepared rapid gradient echo sequence, TR = 2300 ms, TE = 4.21 ms, 1.1 mm slice thickness, 900 ms inversion time, 9° flip angle).

Image analysis was performed using the statistical parametric mapping software SPM8 (Wellcome Trust Centre for Neuroimaging, University College London, United Kingdom) implemented in MATLAB (Mathworks Inc., Natick, USA). Preprocessing included correction for slice-timing differences, realignment to the first image to adjust for movement, segmentation, normalization to standard MNI space (at isotropic voxel size), and smoothing with a Gaussian kernel of 8 mm FWHM. The first level (individual subject) analyses were set up using the general linear model approach, with events of interest being modeled by regressors.

The following events were modeled by separate regressors (feedback onset was chosen as onset of the HRF): (a) correct responses, with concurrent positive feedback; (b) probabilistic errors; (c) final reversal errors and (d) other preceding reversal errors (later termed “other errors”), which were errors following a contingency reversal, but which were not final reversal errors (i.e., which were not followed by a switch to the correct response in the subsequent trial). The interstimulus interval, during which a black fixation cross on white background was presented, served as an implicitly modeled affectively neutral baseline. Based on these regressors, and as recommended by previous work (Cools et al., 2002, 2006), the following contrasts were computed for each session: (1) final errors minus correct responses (FE vs. CR), as a measure of reversal-related neural processes which were assumed to be most prevalent during such final negative feedback. This was the main contrast of interest because it assesses the point at which subjects learn to reverse their response and has been the focus in previous fMRI studies on reversal learning (e.g., Cools et al., 2002) (2) final errors minus other errors (FE vs. OE) which assesses the processes specific to learning to reverse while controlling for the effects of negative feedback. Thus, we compare negative feedback trials (FE) vs. other negative feedback trials (OE). One more contrast was (3) All errors minus All correct responses which assesses the processing of negative feedback in general. Additionally (4), we modeled (FE vs. Baseline), (OE vs. Baseline), and (CR vs. Baseline) contrasts. Contrast images of these regressors from the first level were then entered into second level random effects analysis, and *t*-tests for independent samples were performed to assess the difference in activity between the HH and LL genotype groups.

The statistical threshold of these analyses was set to *P* = 0.05, FWE-corrected at the voxel-level. Anatomical labeling was performed according to the WFU-PickAtlas (Version 3.3, Wake Forest University, School of Medicine, Winston-Salem, North Carolina). For the cingulate cortex, we followed Vogt's nomenclature (Vogt, 2005).

### ROI analyses

**Figure 2:**
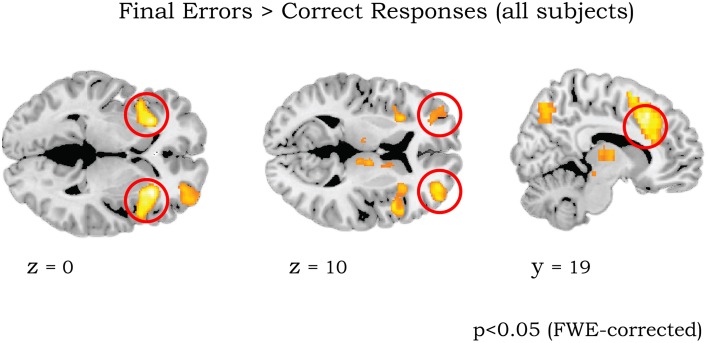
**Whole brain activation of all 43 participants for contrast Final Errors vs. Correct Responses (threshold** ***p*** **< 0.05 FWE corrected at voxel level)**. The clusters in left/right anterior insula/ventrolateral PFC (circled in left panel), left/right lateral OFC (middle panel) and aMCC (right panel) were used for the ROI analyses (see Methods).

Our primary regions of interests (ROIs) were lateral OFC, aMCC, aInsula/VLPFC, and striatum. These areas have not only been systematically been involved in reversal learning, but also have a documented high level of dynorphins expression in humans (Hurd, 1996; Cools et al., 2002; Cools, 2006; Dodds et al., 2008). For statistical analysis, ROIs for left/right aInsula/VLPFC, left/right lateral OFC, and aMCC were defined as spheres of 8 mm radius, with the centers defined by the peak of activation found with the contrast FE > CR from all subjects (coordinates MNI x/y/z = −32 24 0/30 24 0, −33 58 5/32 51 10, and 9 19 33, respectively, see Results and Figure 2). Importantly, these coordinates are also similar to the activation for the same contrast in the original studies by Cools et al. (2002, 2006).

To create ROIs for different subdivisions of the striatum (caudate, putamen, and ventral striatum), we used the anatomical templates for left, right putamen and left, right caudate from the WFU-PickAtlas (Version 3.3, Wake Forest University, School of Medicine, Winston-Salem, North Carolina; www.ansir.wfubmc.edu). The masks for the left and right ventral striatum were defined in the same way as our previous dynorphins study (Votinov et al., 2014) by a conjunction of the “caudate head” template provided in the WFU-PickAtlas and the “accumbens” template taken from the Harvard–Oxford Subcortical Structural Atlas which is implemented in FSL software.

Mean parameter estimates within 11 ROI masks (2 × Caudate, 2 × Putamen, 2 × VS, 2 × lOFC, 2 × aInsula/VLPFC, and aMCC) were extracted, by using the MATLAB-based REX tool (MIT, web.mit.edu/swg/software.htm), for the contrasts (FE vs. CR), (FE > OE), (All errors vs. All Correct Responses) and (FE vs. Baseline), (OE vs. Baseline), (CR vs. Baseline) from each individual, and entered into statistical analysis. Group differences for each contrast were analyzed in SPSS 20.0 (SPSS Inc., Armonk, USA) using a MANOVA with genotype groups (LL, HH) as between-subjects factors. This procedure was used according to the recommendations from a recent statistics and data analysis textbook (Field, 2013). Consisting of several steps, the analysis demonstrates in the 1st step (MANOVA) that there is a main effect of genotype on all ROIs. This “overall” finding is then explored in more details, in the second step (univariate ANOVAs), which shows which ROIs specifically drive the MANOVA main effect. The last step, i.e., Bonferroni corrected *post-hoc t*-tests (automatic correction by SPSS), are then used to demonstrate the directions of these differences. In addition, we report: V (Pillai–Barlett trace), which is an alternative indicator of significance than using the *F*-value; Eta-squared (η^2^) which is a measure of effect size and observed power (α).

If the sphericity assumption was violated (significant results in Mauchly's test of sphericity), degrees of freedom were corrected using Greenhouse-Geisser estimates of sphericity. Significance was evaluated at *P* < 0.05. All data are reported as means ± SE. Additionally we performed exploratory correlation analysis (Pearson) between the extracted parameter estimates from left lateral OFC (the only ROI where activation was significantly different between the groups; see Results) in the contrast Final errors vs. Correct responses and the numbers of perseverative errors for each genotype group.

### Effective connectivity analysis

Based on the significant results from the ROI and whole-brain functional segregation analyses (see Results), we explored whether the groups also differed with respect to effective connectivity between left lateral OFC with other parts of the brain. The seed region of this analysis was based on the same sphere with 8 mm diameter in left lateral OFC from the ROI analysis (i.e., with the center defined by the contrast FE vs. CR from all subjects).

Analyses were implemented within the framework of psychophysiological interaction (PPI) analyses. Using a spherical seed region, we calculated the PPI term as the product of the mean time course in this region and the respective psychological variable, namely “reversal learning process,” defined as the contrast FE > CR. All three variables (time course in seed region, psychological variable, and interaction term) were entered into a new general linear model for each subject. The way PPI analysis is implemented in the SPM software package, it is not possible to perform PPI preprocessing on 2 sessions simultaneously. We therefore performed two PPI analyses for each run and then jointly analyzed them using the factorial design. Full factorial 2 (genotype) × 2 (fMRI sessions) ANOVA models were set up to compare the parameter estimates of the PPI term between the HH and LL genotype groups. We reported results, which survived only after small volume correction within ROI masks (threshold *p* = 0.05).

## Results

### Behavioral data

Analysis of consumptions of alcohol, coffee, tobacco and energy drinks did not reveal any significant differences between genotype groups [*F*_(1, 41)_ = 0.47, *p* = 0.49, partial η^2^ = 0.011, α = 0.1] and no interaction effect for genotype^*^type of substance [*F*_(1, 41)_ = 0.073, *p* = 0.78, partial η^2^ = 0.002, α = 0.058] (see Table 2).

We observed a significant main effect of genotype for errors [*F*_(1, 41)_ = 5.09, *p* = 0.029, partial η^2^ = 0.11, α = 0.59]. Followed up *post-hoc* comparison demonstrated that the HH group made more perseverative errors (mean ± SEM: 27.68 ± 1.7) than the LL group (21.23 ± 1.7), but no difference for spontaneous errors was found.

There was no main effect of genotype for reaction time data [*F*_(1, 41)_ = 0.04, *p* = 0.84, partial η^2^ = 0.001, α = 0.054] and no interaction genotype^*^type of feedback *p* = 0.48) (see Table 3).

**Table 3:** **Behavioral results**.

	**Mean ± SEM**	***P*-value**	**Partial Eta squared**	**Observed power**
Number of perseverative errors	LL 21.68 ± 1.6	0.03	0.11	0.59
	HH 27.24 ± 1.6			
Number of spontaneous errors	LL 4.1 ± 1.1	0.35	0.021	0.15
	HH 5.6 ± 1.2			
Response latency following misleading errors	LL 515.44 ± 20.4	0.68	0.004	0.07
	HH 503.39 ± 20.8			
Response latency following correct responses	LL 491.83 ± 18.5	0.76	0.002	0.06
	HH 500.0 ± 19			
Response latency following final reversal errors	LL 511.70 ± 25	0.72	0.003	0.06
	HH 498.86 ± 25.6			

There was also no significant main effect of genotype on the scores of the BIS11 [*F*_(1, 41)_ = 0.6, *p* = 0.44, partial η^2^ = 0.01, α = 0.11] and BIS/BAS [*F*_(1, 41)_ = 1.71, *p* = 0.2, partial η^2^ = 0.04, α = 0.24] questionnaires (Table 4), and no significant interactions genotype^*^subscales (all *p* > 0.38).

**Table 4:** **Results for BIS/BAS and BIS11 scale**.

**Genotype groups**	**BIS scale**	**BAS drive**	**BAS fun seeking**	**BAS reward responsiveness**	**BIS11 attentional impulsiveness**	**BIS11 motor impulsiveness**	**BIS11 non-planning impulsiveness**
LL	3.01 ± 0.12	3.12 ± 0.09	3.11 ± 0.1	3.41 ± 0.09	16.4 ± 0.6	23.5 ± 0.8	22.18 ± 1
HH	2.88 ± 0.12	3.03 ± 0.1	2.95 ± 0.1	3.30 ± 0.09	16.5 ± 0.6	23.9 ± 0.8	23.8 ± 1

*All p > 0.26*.

### fMRI data for the whole sample

The initial analyses were applied to the whole sample without taking into account the genotype groups, as their rationale was to determine whether our results are consistent with those of previous studies (Cools et al., 2002, 2006). The comparison of final reversal errors with correct responses after applying a threshold of *p* < 0.05 (FWE corrected at voxel level) revealed activation in right striatum, bilateral aInsula/VLPFC, bilateral lateral OFC, perigenual ACC, anterior mid-cingulate cortex (MCC), thalamus, and midbrain (Figure 2 and Table 5), which was very much in line with previous reports.

**Table 5:** **List of clusters with significant activation for the contrast Final Errors vs. Correct Responses (thresholded at** ***p*** **< 0.05, FWE corrected at voxel level) of all 43 participants**.

**Region**	**L/R**	**Cluster size**	***T***	***x***	***y***	***z***	***p*-Value**
aInsula/Ventrolateral prefrontal cortex	R	5388	13.21	34	24	0	<0.0001
Mid-cingulate cortex		s.c.	12.27	9	23	43	<0.0001
Mid-cingulate cortex		s.c.	12.17	4	21	38	<0.0001
perigenual anterior cingulate cortex		s.c.	9.09	8	28	24	<0.0001
Medial frontal gyrus		s.c.	11.56	2	15	48	<0.0001
Superior frontal gyrus	R	s.c.	10.45	10	9	58	<0.0001
Dorsolateral prefrontal cortex	R	s.c.	10.08	39	26	34	<0.0001
Lateral orbitofrontal cortex	R	s.c.	8.77	32	51	10	<0.0001
Lateral orbitofrontal cortex	R	s.c.	7.48	18	54	−14	<0.0001
aInsula/Ventrolateral prefrontal cortex	L	471	12.03	−30	24	0	<0.0001
		s.c	10	−40	15	−5	<0.0001
Inferior parietal lobule	R	1282	11.8	33	−48	43	<0.0001
		s.c.	9.81	51	−48	52	<0.0001
Inferior parietal lobule	L	484	9.57	−34	−45	38	<0.0001
		s.c.	7.99	−43	−40	43	<0.0001
SMA	L	185	8.4	−25	−3	52	<0.0001
Precuneus	R	343	7.9	10	−72	53	<0.0001
	L	s.c.	7.2	−4	−74	43	<0.0001
Lateral orbitofrontal cortex	L	232	7.6	−33	58	5	<0.0001
	L	s.c	7.0	−28	48	14	<0.0001
Middle temporal gyrus	R	114	7.7	50	−28	−10	<0.0001
Thalamus	R	76	7.38	10	−9	10	<0.0001
		s.c.	6.93	9	−20	10	<0.0001
Striatum	R	32	6.74	12	4	5	<0.0001
Lateral orbitofrontal cortex	L	16	6.53	−16	46	−19	<0.0001
Midbrain		6	6.2	9	−24	−10	0.003
Thalamus	L	7	6.16	−10	−15	10	0.002

*L/R, left/right in the brain; s.c., sub-cluster; SMA, Supplementary Motor area*.

### Effect of genotype on activation during reversal learning—ROI analysis

In the second step we analyzed differences between genotype groups in *a priori* defined areas of interest, using an ROI approach.

#### Final errors vs. correct responses

**Figure 3:**
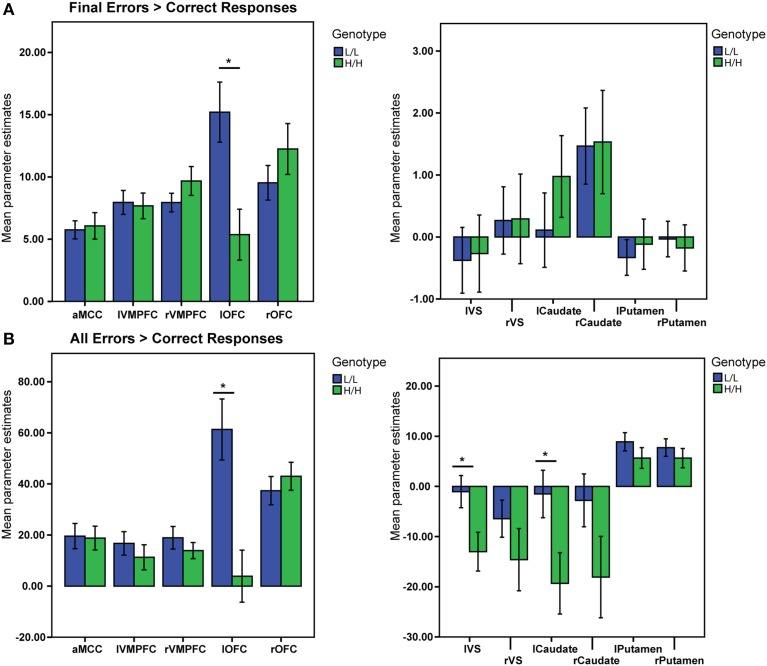
**BOLD signal (Parameter Estimates ± SEM) from all regions of interests in different (68-bp VNTR) prodynorphin promoter polymorphism genotypes (LL- and HH-alleles). (A)** Final Errors vs. Correct Responses contrast. The HH (“high level pDYN expression”) group shows significantly lower activation in the left lateral OFC compared to the LL (“low level pDYN expression”) group; **(B)** All Errors vs. All Correct Responses. The HH group shows lower activation in the left ventral striatum, left caudate, and left lateral OFC compared to the LL group (^*^p < 0.05).

There was a significant main effect of genotype on the activation in the ROIs, V (Pillai's trace) = 0.9, [*F*_(11, 31)_ = 3.26, *p* = 0.005, partial η^2^ = 0.53, observed power α = 0.96]. Univariate ANOVAs revealed an effect of genotype on activation in left lateral OFC [*F*_(1, 41)_ = 9.5, *p* = 0.004, partial η^2^ = 0.19, α = 0.85]. Bonferroni-corrected *post-hoc* comparisons demonstrated significantly higher activation for the LL group than for the HH group in left lateral OFC (LL: 15.2 ± 2.17 arbitrary units; HH: 5.4 ± 2.23 a.u., *p* ≥ 0.004; see Figure 3A), but not in the other ROIs (all *p* ≥ 0.21).

#### Final errors vs. other errors

There was no significant main effect of genotype on activation for this contrast in all ROIs *V* = 0.35, [*F*_(11, 31)_ = 1.5, *p* = 0.16, partial η^2^ = 0.35, α = 0.65].

#### All errors vs. all correct responses

There was a significant main effect of genotype on ROI activations, *V* = 0.5, [*F*_(11, 31)_ = 2.83, *p* = 0.011, partial η^2^ = 0.5, α = 0.92]. Separate univariate ANOVAs revealed an effect of genotype on activation in left VS [*F*_(1, 41)_ = 5.71, *p* = 0.021, partial η^2^ = 0.12, α = 0.64], left Caudate [*F*_(1, 41)_ = 5.35, *p* = 0.026, partial η^2^ = 0.11, α = 0.61], and left OFC [*F*_(1, 41)_ = 13.29, *p* = 0.001, partial η^2^ = 0.24, α = 0.94]. Bonferroni-corrected *post-hoc* comparisons demonstrated significantly higher activity for the LL group than for the HH group in left VS (LL: −1.04 ± 3.4 arbitrary units; HH: −13.01 ± 3.5 a.u., *p* = 0.021), left Caudate (LL: −1.5 ± 5.3 a. u.; HH: −19.31 ± 5.5 a.u., *p* = 0.026), and left lateral OFC (LL: 61.32 ± 11 a. u.; HH: 3.8 ± 11.2 a.u., *p* ≥ 0.001), but not in the other ROIs (all *p* ≥ 0.11; see Figure 3B).

#### Final errors vs. baseline

**Figure 4:**
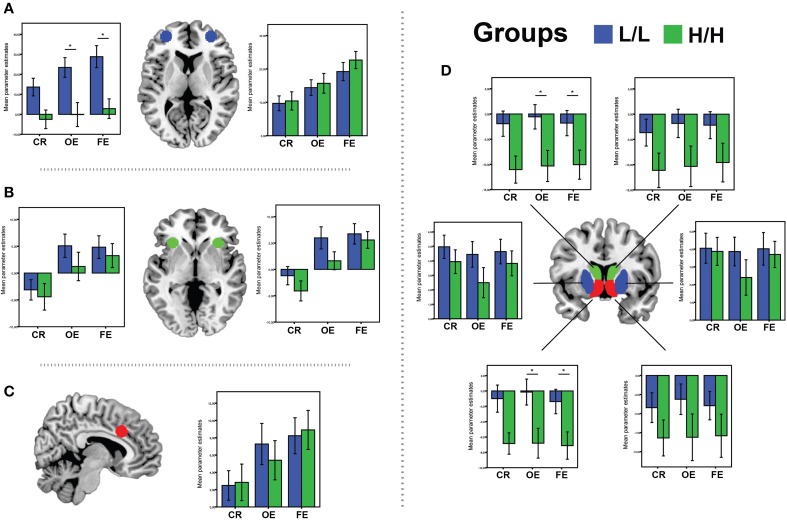
**BOLD signal (Parameter Estimates ± SEM) from all regions of interests in different (68-bp VNTR) prodynorphin promoter polymorphism genotypes (LL- and HH-alleles) for CR (Correct Response vs. Baseline), OE (Other Errors vs. Baseline), and FE (Final Errors vs. Baseline) in: (A) bilateral OFC; (B) bilateral aInsula/VMPFC; (C) aMCC; (D) Caudate (green), Ventral Striatum (red) and Putamen (blue)**. ^*^Represents significant difference (*p* < 0.05), only for the contrast where was significant main effect of genotype on ROI activations.

There was a significant main effect of genotype on ROI activations, *V* = 0.5, [*F*_(11, 31)_ = 2.8, *p* = 0.01, partial η^2^ = 0.5, α = 0.93]. Separate univariate ANOVAs revealed an effect of genotype on activation in left VS [*F*_(1, 41)_ = 5.8, *p* = 0.02, partial η^2^ = 0.12, α = 0.65], left Caudate [*F*_(1, 41)_ = 4.6, *p* = 0.037, partial η^2^ = 0.1, α = 0.56], and left OFC [*F*_(1, 41)_ = 12.35, *p* = 0.001, partial η^2^ = 0.23, α = 0.92]. Bonferroni-corrected *post-hoc* comparisons demonstrated significantly higher activity for the LL group than for the HH group in left VS (LL: −1.38 ± 1.6 arbitrary units; HH: −7.12 ± 1.7 a.u., *p* = 0.02), left Caudate (LL: −1.8 ± 2.6 a. u.; HH: −10.04 ± 2.7 a.u., *p* = 0.037), and left lateral OFC (LL: 28.82 ± 5.1 a. u.; HH: 2.84 ± 5.2 a.u., *p* = 0.001), but not in the other ROIs (all *p* ≥ 0.12; see Figure 4).

#### Other errors vs. baseline

There was a significant main effect of genotype on ROI activations, *V* = 0.45, [*F*_(11, 31)_ = 2.38, *p* = 0.028, partial η^2^ = 0.45, observed power α = 0.86]. Separate univariate ANOVAs revealed an effect of genotype on activation in left VS [*F*_(1, 41)_ = 6.72, *p* = 0.013, partial η^2^ = 0.14, α = 0.71], left Caudate [*F*_(1, 41)_ = 6.2, *p* = 0.017, partial η^2^ = 0.13, α = 0.68], and left OFC [*F*_(1, 41)_ = 9.3, *p* = 0.004, partial η^2^ = 0.18, α = 0.84]. Bonferroni-corrected *post-hoc* comparisons demonstrated significantly higher activity for the LL group than for the HH group in left VS (LL: −0.14 ± 1.7 arbitrary units; HH: −6.8 ± 1.8 a.u., *p* = 0.013), left Caudate (LL: −0.5 ± 2.7 a. u.; HH: −10.3 ± 2.7 a.u., *p* = 0.017), and left lateral OFC (LL: 23.44 ± 5.3 a. u.; HH: −0.12 ± 5.5 a.u., *p* = 0.004), but not in the other ROIs (all *p* ≥ 0.11; see Figure 4).

#### Correct responses vs. baseline

There was no significant main effect of genotype on ROI activations, *V* = 0.45, [*F*_(11, 31)_ = 1.5, *p* = 0.16, partial η^2^ = 0.35, observed power α = 0.65].

### Effective connectivity of left OFC and reversal learning

Based on the finding that left lateral OFC had shown a significant difference between genotype groups in the contrast Final Error vs. Correct responses, this area was used as a seed region for a follow-up exploratory connectivity analysis. After applying small volume correction (ROI masks), the LL group, compared to the HH group, had stronger functional connectivity of left lateral OFC only with aMCC (−9 6 46, *T* = 4.06, *p* = 0.009), left aInsula /VLPFC (−36 20 5, *T* = 3.48, *p* = 0.025), right VLPFC/aInsula (34 28 5, *T* = 3.22, *p* = 0.029 during Final Errors vs. Correct responses.

### Correlation analyses

#### Contrast: final errors vs. correct responses

The results for the whole group of subjects demonstrated that activation in left lateral OFC was significantly negatively correlated with the number of perseverative errors (*r* = −0.43, *p* = 0.004). The same correlation analysis separately for each genotype group showed that activation in left lateral OFC correlated significantly negatively with the number of perseverative errors (*r* = −0.51, *p* = 0.017) for the HH group, but not for the LL group (*r* = −0.14, *p* = 0.52; correlation differed trend-like, *p* = 0.09).

## Discussion

The current study is the first to demonstrate that genetically driven modulation of the endogenous dynorphin opioid peptides affects performance in a probabilistic reversal learning task. Group-independent findings of the whole-brain analyses showed that reversal learning resulted in higher activity in the striatum, aInsula/VLPFC, thalamus, aMCC, and lateral orbitofrontal cortex, which is in line with numerous animal and human brain studies on reversal learning (Duncan, 2001; Cools et al., 2002; Fellows and Farah, 2003; Kringelbach and Rolls, 2003; Clark et al., 2004; Hornak et al., 2004). The HH genotype group made significantly more reversal errors than the LL genotype group, implying that the HH group is less efficient in adapting their behavior. These behavioral findings were accompanied by differences in BOLD signal changes and effective connectivity. The LL group displayed significantly higher activation in the left lateral OFC during final reversal errors, i.e., immediately before shifting their responses to adapt to a new, correct pattern. In addition, the LL group displayed significantly higher activation in left lateral OFC, left VS, and left caudate during the processing of errors in general. Moreover, this group had stronger connectivity of left lateral OFC with aMCC, mOFC, and bilateral VLPFC/aInsula than the HH group during final reversal errors.

The ROI analysis revealed that activity in the left lateral OFC was significantly different between genotype groups during processing final reversal errors and other types of feedback. These findings are in line with various studies demonstrating important contributions of orbitofrontal cortex in reversal learning (Chudasama and Robbins, 2003; O'Doherty et al., 2003; Hornak et al., 2004; Hampshire and Owen, 2006; Tsuchida et al., 2010). Similarly, patients and monkeys with lesions in the OFC are impaired in reversal learning and insensitive to negative outcomes (Berlin et al., 2004; Hornak et al., 2004; Clarke et al., 2008). fMRI studies suggested that the lateral and medial OFC differentially encode rewards, with lateral OFC processing negative rewards and medial OFC processing positive rewards, respectively (see Kringelbach, 2005 for review). Together, these findings suggest that (lateral) orbital prefrontal cortex monitors and decodes changes in reward contingencies and uses this information to guide behavior in the task (Rolls, 2004). In the present study, the lower the activation was in the OFC of the HH group, the more perseverative errors they made. This indicates that the HH group was less able to adapt their behavior based on feedback. This interpretation is also supported by the lower activation in the left lateral OFC of the HH group than the LL group for the contrasts of Final errors vs. Baseline and Other Errors vs. Baseline. Findings in these two contrasts demonstrate that the effect of the comparison FE vs. CR is not driven just by a difference in the numbers of FE and CR trials.

Furthermore, the HH group demonstrated reduced functional connectivity of left lateral OFC with aMCC and bilateral aInsula/VLPFC when processing final reversal errors.

It has been shown that aMCC may help to guide behavior by integrating information from its own “internal network of cells” and then directly and/or indirectly influence attention allocation, motor preparation, and motor responses (Bush et al., 2002). In addition, a more recent reversal learning study demonstrated that this region is playing a key role in implementing the behavioral decision itself (Hampton and O'Doherty, 2007). Shenhav with colleagues proposed that aMCC (dACC in their notion) integrates the expected payoff from a controlled process, the amount of control that must be invested to achieve that payoff, and the cost in terms of cognitive effort (Shenhav et al., 2013). Activation in bilateral VLPFC/anterior insula is also constantly observed in reversal learning studies and has been suggested to also play a key role in the adaptation of behavior in response to changes (Cools et al., 2002, 2006; Hampton et al., 2006). A new study by Rudebeck et al. (2013) highlighted the importance of fibers which connect the OFC with other brain regions associated with behavioral flexibility. They demonstrated that a fiber-sparing lesion of the OFC in monkeys led to impaired updating of reward value, but did not influence behavioral flexibility or emotional regulation. However, when they applied damage to the fibers passing near or through the OFC, it caused an impairment of behavioral flexibility (Rudebeck et al., 2013). This is in line with our previous PDYN study (Votinov et al., 2014) which had also demonstrated a difference between genotype groups in functional connectivity between OFC and other regions.

Therefore, our data suggest that the decreased connectivity of lateral OFC with aMCC and aInsula/VLPFC in the HH group during the processing of final errors may be related to the tendency to maintain the previous task-set due to a lack of updating information that reward contingencies have changed.

One possible mechanistic interpretation of the inferior performance of the HH group in reversal learning is that dynorphins modulate dopamine levels in the striatum and prefrontal cortex (Di Chiara and Imperato, 1988b; Steiner and Gerfen, 1998; Margolis et al., 2006; Nestler and Carlezon, 2006). Microdialysis studies showed that administration of selective k-opioid receptor (KOR) agonists decreased dopamine overflow in the nucleus accumbens and dorsal striatum (Di Chiara and Imperato, 1988a). Similar effects on dopamine levels were observed after intra-striatal perfusion of dynorphins (Zhang et al., 2004). Furthermore, selective KOR agonists may increase dopamine reuptake (Shippenberg et al., 2001). One recent fMRI study on alert primates showed that opioid μ and *k* agonists can modulate brain activation particularly in left nucleus accumbens and caudate (Kaufman et al., 2013), which was also the case in our study for the contrasts Final errors vs. baseline and Other errors vs. baseline. Furthermore, similar to the effect on the striatum, KOR agonists also affect prefrontal cortex by decreasing dopamine overflow (Margolis et al., 2006; Tejeda et al., 2012). Our previous dynorphins study (Votinov et al., 2014) also demonstrated differences in the brain activation for medial orbitofrontal cortex between HH and LL genotype groups. In that study we observed that HH group had higher activation in mOFC than the LL group in response to upcoming monetary reward, which interpreted as increased reward sensitivity in the HH group. Increased reward sensitivity may also partially explain the higher number of perseverative errors in HH group, because they “stick” to the previous positively rewarded feedback and do not adjust their response accordingly.

To sum up, substantial evidence from pharmacological and genetic studies suggests that the dynorphins/KOR system regulates the basal activity of dopamine neurons in the cortico-striatal circuit (for review see Shippenberg et al., 2007) which may lead to an impairment of the mechanisms involved in behavioral adaptation during the reversal task.

As to the role of dopamine for reversal learning, animals with dopaminergic lesions of the prelimbic cortex failed to adapt their instrumental response to changes in contingency. Similarly, microinfusions of the dopamine D1/D2 receptor antagonist flupenthixol in the prelimbic cortex led to animals failing to adapt their response to changes in contingency (Naneix et al., 2009), as did genetic deletion of D2 receptors (Kruzich and Grandy, 2004). A human study of the DRD2/ANKK1-TaqIa polymorphism demonstrated that the A1+ group, which is associated with reduced expression of dopamine D2 receptors, showed reduced recruitment of the right ventral striatum and the right lateral orbitofrontal cortex during reversals (Jocham et al., 2009). Along these lines, A1-allele carriers with reduced dopamine D2 receptor densities learned to avoid actions with negative consequences less efficiently, which led the authors to conclude that learning from errors requires dopaminergic signaling (Klein et al., 2007). Thus, these data indicate that dopamine signaling in the functional PFC–striatal circuit is critical for processing punishments and flexibly guiding decision making (Clarke et al., 2008). Our results suggest that dynorphins may interfere with these processes.

However, the effect of dynorphins may also work via the serotonin system, as both the dopamine and serotonin system affect reversal learning (Groman et al., 2013). Indeed, numerous lesion, genetics and behavioral animal studies confirmed the role of serotonin depletion in performance impairment in reversal learning tasks (Clarke et al., 2007; Izquierdo et al., 2007; Izquierdo and Jentsch, 2012; Rygula et al., 2014). A recent human behavioral study by den Ouden et al. (2013) demonstrated a differential role in reversal learning for two gene polymorphism (SERT and DAT1) which encode dopamine and serotonin transporters. They observed that the SERT polymorphism altered behavioral adaptation after losses, while DAT1 genotype affected the influence of prior choice on perseveration. Since dynorphins have been found to mainly influence dopamine release and dopamine reuptake (see review Shippenberg et al., 2001) and our study found significant differences in the numbers of perseverative errors, we may speculate that the 64 bp dynorphins polymorphism had similar effects on perseverative behavior as described in the paper from den Ouden and colleagues. However, it should be noted, that they used many more participants as well as a different version of probabilistic reversal task.

A limitation of our study is that the fMRI method we used did not allow for any direct measures of changes in neither opioidergic, dopaminergic, or serotonergic function. In combination with very recent conflicting evidence on how the different PDYN genotypes are related to dynorphins levels and potency (Zimprich et al., 2000; Nikoshkov et al., 2008; Babbitt et al., 2010; Rouault et al., 2011), we therefore advocate future studies including pharmacological manipulations and positron emission tomography to clarify by which neurochemical mechanisms genotypic variation is affecting reversal learning.

As to the role of OFC in reversal learning, a resent review by Stalnaker et al. (2015) about possible functions of OFC in response inhibition, outcome association, emotions, reward valuation and error prediction suggests that these are not core functions of OFC, but that it plays a more complex role (Stalnaker et al., 2015). They proposed that OFC provides state information (cognitive map) which is then used by other areas, therefore playing the role of an integrator.

Thus, it is possible that the changes in neuromodulatory activity and effective connectivity of the OFC associated with the PDYN polymorphism result in changes in several functions, including reward evaluation and response inhibition.

## Conclusions

The results of this study highlight the role of the PDYN functional polymorphism in reversal learning. Our findings demonstrate that individuals with the HH genotype are less efficient to flexibly modify behavior than individuals with the LL genotype. On a neural level, the HH group demonstrated less engagement of the cortico-striatal circuitry and less interaction between lateral OFC and aMCC, aInsula/VLPFC regions during the reversal stage. The impairment of the HH genotype group may therefore relate to a lack of connectivity lateral OFC with aMCC, aInsula/VLPFC regions and a lack of monitoring changes in reward contingency. Overall, these findings provide first evidence that the opioid system may contribute to individual differences in reversal learning.

### Conflict of interest statement

The authors declare that the research was conducted in the absence of any commercial or financial relationships that could be construed as a potential conflict of interest.
